# Apoptosis through Bcl-2/Bax and Cleaved Caspase Up-Regulation in Melanoma Treated by Boron Neutron Capture Therapy

**DOI:** 10.1371/journal.pone.0059639

**Published:** 2013-03-20

**Authors:** Fernanda Faião-Flores, Paulo Rogério Pinto Coelho, João Dias Toledo Arruda-Neto, Silvya Stuchi Maria-Engler, Manoela Tiago, Vera Luiza Capelozzi, Ricardo Rodrigues Giorgi, Durvanei Augusto Maria

**Affiliations:** 1 Laboratory of Biochemistry and Biophysics, Butantan Institute, São Paulo, Brazil; 2 Faculty of Medicine, University of São Paulo, São Paulo, Brazil; 3 Institute for Nuclear and Energy Research, São Paulo, Brazil; 4 Physics Institute, University of São Paulo, São Paulo, Brazil; 5 CEPESq/UniÍtalo – Italy-Brazilian University Center, São Paulo, Brazil; 6 Department of Clinical Chemistry & Toxicology, School of Pharmaceutical Sciences, University of São Paulo, São Paulo, Brazil; 7 Laboratory for Cellular and Molecular Endocrinology (LIM-25) School of Medicine, University of São Paulo, São Paulo, Brazil; 8 Santo Amaro University UNISA, São Paulo, Brazil; University of Tennessee, United States of America

## Abstract

Boron neutron capture therapy (BNCT) is a binary treatment involving selective accumulation of boron carriers in a tumor followed by irradiation with a thermal or epithermal neutron beam. The neutron capture reaction with a boron-10 nucleus yields high linear energy transfer (LET) particles, alpha and ^7^Li, with a range of 5 to 9 µm. These particles can only travel very short distances and release their damaging energy directly into the cells containing the boron compound. We aimed to evaluate proliferation, apoptosis and extracellular matrix (ECM) modifications of B16F10 melanoma and normal human melanocytes after BNCT. The amounts of soluble collagen and Hsp47, indicating collagen synthesis in the ECM, as well as the cellular markers of apoptosis, were investigated. BNCT decreased proliferation, altered the ECM by decreasing collagen synthesis and induced apoptosis by regulating Bcl-2/Bax in melanoma. Additionally, BNCT also increased the levels of TNF receptor and the cleaved caspases 3, 7, 8 and 9 in melanoma. These results suggest that multiple pathways related to cell death and cell cycle arrest are involved in the treatment of melanoma by BNCT.

## Introduction

Boron neutron capture therapy (BNCT) is based on the nuclear capture and fission reactions of the ^10^B nucleus with low energy thermal/epithermal neutrons to yield high linear energy transfer α particles and recoiling ^7^Li nuclei. Since the path lengths of the particles are approximately 9 to 10 µm, equal to the dimensions of a single cell, ^10^B-containing cells are selectively destroyed by BNCT [1].

Boronophenylalanine (BPA), an analog of tyrosine, has been utilized as a boron drug for BNCT, accumulating at higher levels in tumors than in normal tissue [2]. Tumor cells selectively uptake BPA, which particularly accumulates in their nuclei and is clinically used in BNCT [1].

In several types of tumors, such as glioblastoma and melanoma, where treatment is usually ineffective in controlling the disease, this approach is potentially beneficial [3]. In recent years, the incidence and mortality of melanoma, a highly invasive and metastatic tumor, has increased [4]. It is the most aggressive form and the cause of a majority of deaths among skin cancer patients [5].

There are few published results about the effects of BNCT on normal melanocytes compared to melanoma cells [6]. These data are extremely important in balancing the effectiveness of BNCT against its side effects on healthy tissues. Some mechanisms involved in damaging the tumor as a result of BNCT are still unknown. This work aimed to understand the mechanism by evaluating proliferation, changes in the extracellular matrix (ECM) and apoptosis after BNCT treatment in melanoma, as well as its putative side effects on normal melanocytes.

## Materials and Methods

### Cell Lines and Culture Conditions

B16F10 murine melanoma cells were purchased from the American Type Culture Collection (CRL-6475) (Manassas, VA). These cells are widely used as a model to study human melanoma because they share many similar characteristics with this cancer type [7]. The cells were cultivated in 75 cm^2^ flasks with DMEM (Cultilab, Brazil) supplemented with 10% inactivated fetal bovine serum (FBS) (Cultilab), 2 mM L-glutamine (Sigma Chemical Company, USA) and 0.1 g/mL streptomycin (FontouraWyeth AS, USA). Primary cultures of skin cells (melanocytes) were obtained from the foreskins of patients at University Hospital (Hospital Universitário – HU-USP). The project was reviewed and approved by the Ethics Committee of HU (HU no. CEP Case 943/09). Patients who donated the cells for use in primary culture consented to this and the terms are documented under number: 943/09. Participants provided written informed consent to participate in this study and the ethics committees approved this consent procedure. The melanocytes were maintained in 254CF medium (SKU # M-500-254CF; Cascade Biologics, USA) supplemented with human melanocyte growth supplement (HMGS – SKU # S-002-5; Cascade Biologics, USA), as previously described [8]. Cells were grown at 37°C in a 5% CO_2_ humidified atmosphere.

### Boronophenylalanine (BPA)

^10^B-enriched (>99%) BPA was purchased from KatChem and converted into a fructose 1∶1 complex to increase its solubility [9].

### Cell Treatment and BNCT Irradiation for Soluble Collagen Quantification and Flow Cytometric Tests

Melanocytes and B16F10 melanoma cells were seeded in 24-well plates at a concentration of 10^5^ cells/mL and allowed to grow for 24 h. B16F10 melanoma cells were treated with 3.3 mg/mL of BPA in all flow cytometric tests (this value is equivalent to 172.0 µg ^10^B/mL). This concentration corresponded to the Inhibitory Concentration of 50% (IC_50_) for this compound in this cell line [10]. Melanocytes were treated with 34.4 mg/mL of BPA in all flow cytometric tests (this value is equivalent to 1.8 mg ^10^B/mL) [6], which corresponded to the IC_50_ for this compound in this cell line. After 90 min of incubation with BPA, the cells were irradiated at the BNCT research facility at the Nuclear and Energetic Research Institute (IPEN, Brazil) [11] for 30 min, using the IEA-R1 nuclear reactor operating at a power of 3.5 MW. The thermal neutron flux, epithermal neutron flux and fast neutron flux at the irradiation position were (2.31±0.03)x10^8^, (4.60±0.10)x10^6^ and (3.50±0.10)×10^7^ n/cm^2^s, respectively. The rate of gamma dose in the air at the irradiation site was 3.50±0.80 Gy h^−1^. Before irradiation, the BPA-enriched incubation medium was removed and the cells were washed in 0.9% saline solution. Another cell group was irradiated without BPA (beam only) and was designated the “irradiated control”. A non-irradiated and without BPA group were also studied and designated “control”.

### Soluble Collagen Quantification by Picrosirius Assay

Picrosirius assays evaluate the quantity of collagen in a sample [12]. The dyes used for this test react specifically with basic groups in the collagen molecule [13], [14].

After irradiation, plates with the supernatant (metabolized culture medium) of melanoma cells and melanocytes were placed in an incubator at 37°C overnight without lids to dry the contents. Then, saturated Bouin’s solution [12] was added to each well, and the samples were incubated for 1 h at room temperature. The dye was removed and 300 µL of distilled water was added. The plate was dried at room temperature for approximately 2 h. After this period, 200 µL of 0.1% Sirus red dye (Sigma Chemical Company, USA) was added for one hour, protected from light. The dye was removed and 250 µL of 0.01 M HCl was added. After that, the HCl solution was removed and the samples were incubated with 150 µL of 0.1 M NaOH for 30 min. The optical density of the samples was read at 550 nm in a spectrophotometer.

### Protein Expression Quantification by Flow Cytometry

After irradiation, cells in supernatant and adherent cells were pelleted by centrifugation at 1800 rpm for 10 min and incubated with 1 µg of specific anti-Bax, anti-Bad, anti-caspase 8, anti-Bcl2, anti-cytochrome c, anti-Hsp47, anti-TNF receptor (tumor necrosis factor-α receptor) or anti-ki67 antibody, as well as 10 µL of Triton X-100 (0.1%) for permeabilization for 1 h at 4°C. The cells were then incubated with secondary antibody conjugated with Alexa Fluor 488 (Life technologies, USA) for 1 h at 4°C, followed by resuspension of the cells in FACS flow buffer. Cells incubated with FITC-conjugated isotype-specific antibodies were used as negative controls. The samples were analyzed for fluorescence (FL-1 channel) on a Becton Dickinson FACScalibur flow cytometer using the Cell Quest acquisition software. Information about the used flow cytometry antibodies is in Table S1.

### Inoculation of B16F10 Melanoma Cells in Mice

Murine B16F10 cells were cultivated in RPMI-1640 medium supplemented with 10% FBS, 2 mM-Lglutamine, 1 mM sodium pyruvate and 100 IU/ml of penicillin and 100 µg/ml of streptomycin (Invitrogen Inc, USA). Cell suspensions were detached from plates with 0.2% trypsin. After trypsin inactivation with 10% FBS, viable cells were counted by trypan blue dye exclusion. For tumor transfer, 5×10^4^ cells were suspended in 100 µl of phosphate buffered saline (PBS) and injected subcutaneously into the flank regions of mice. Ten to fourteen days after inoculation, the tumors became macroscopically apparent.

### Antitumor Activity: Macroscopy

Mice were inoculated with B16F10 melanoma cells as described above and were randomly allocated to four groups of 5 animals. On day 14 (counted from the initial inoculation date), the BNCT group was intraperitoneally (i.p.) injected with BPA (250 mg/Kg body mass), followed by thermal neutron irradiation. The irradiated control group did not receive BPA, but only the same thermal neutron irradiation. The control group received only i.p. saline solution. Tumor sizes were measured daily using a caliper-like instrument. The size measurement was converted into tumor volume by the equation: tumor volume = length × width^2^/0.52 [15]. Necropsies were performed 15 or 22 days after tumor inoculation (1 or 7 days after irradiation, respectively), according to the method of Dagrosa and co-workers [16]. The mice were euthanized by cervical dislocation and then necropsied. Primary tumors were then resected and processed for histological examination.

This study was carried out in strict accordance with the recommendations in the Guide for the Care and Use of Laboratory Animals of the National Institutes of Health. The protocol was approved by the Ethical Committee for Animal Research at the Butantan Institute (Permit Number: 479/09).

### RNA Extraction

Tumor tissue collected in RNA later (Ambion) was fragmented in a tissue pulverizer (Mikro-Dismembrator U, B. Braun Biotech International, Melsungen, Hesse, Germany). The total mouse RNA was extracted from approximately 100 mg of tissue after homogenization, using the Trizol kit (Invitrogen Corporation, Carlsbad, CA, USA), in accordance with the manufacturer’s recommendations.

### Quantitative Real-time Polymerase Chain Reaction (qRT-PCR) Analysis

Reactions were conducted on a Rotor-GeneTM 6000 (Corbett Research, Sydney, NSW, Australia) using a SuperScriptTM III Platinum® SYBR® Green One-Step qRT-PCR kit (Invitrogen Corporation). The reactions were prepared according to standard protocols. Reaction mixture contained 50 ng of total RNA from each sample, 1.0 µM of each primer, 12.5 µL of 2X SYBR® Green Reaction Mix and 0.5 µL of SuperScriptTM III RT/Platinum® Taq Mix to a final volume of 25 µL. Negative samples were run for each qRT-PCR assay consisting of no RNA in the reverse transcriptase reaction and no cDNA in the PCR. Reactions were carried out under the following cycling conditions: 50°C for 30 min, 95°C for 15 min, and 40 cycles of 95°C for 20 s, followed by 56°C for 30 s and 72°C for 30 s before a final primer sequence extension incubation at 72°C for 5 min. Fluorescence changes were monitored after each cycle, and melting curve analysis was performed at the end of cycles to verify PCR product identity (72°C ramping to 99°C at 0.2°C/second with continuous fluorescence readings). Specificity of amplicons was also ensured by agarose gel electrophoresis to visualize a unique product fragment of appropriate size. Gene-specific primer pairs were located on two adjacent exons to achieve a high level of specificity and to avoid detection of genomic DNA. Primers were designed to have similar GC content and melting temperatures using the Primer3_cgi v 0.2 program.

The nucleotide sequences for the primers were: Caspase 3 (NM_009810.2) forward 5′- TGA CTG GAA AGC CGA AAC TC 3′ and reverse 5′- AGC CTC CAC CGG TAT CTT CTV3′; Caspase 8 (NM_009812.2) forward 5′- CCG AGC TGG ACT TGT GACC -3′ and reverse 5′- CTG CCC AGT TCT TCA GCA AT-3′ and HPRT (NM_013556.2) forward 5′ CTT CCT CCT CAG ACC GCT TT -3′ and reverse 5′- TTT VCCA AAT CCT CGG CAT AA - 3′. Amplified fragment sizes were 122, 118 and 145 bp for Caspase 3, Caspase 8 and HPRT, respectively. To evaluate the amplification efficiency of each target and housekeeping gene (HPRT), standard curves were constructed from a reference sample of RNA using duplicate serial dilutions at five different RNA concentrations (0.8, 4, 20, 100, and 500 ng/µL). qRT-PCR quantification of expression of the target gene in tumor samples was accomplished by measuring the fractional cycle number at which the magnitude of expression reached a fixed threshold (CT = 0.02974) directly related to the amount of PCR product. The mathematical method used was the model described by Pfaffl et al [17], since the amplification efficiencies of the target gene and reference were not equivalent.

### Tissue Preparation and Immunohistochemical Analysis

All tumor tissues were fixed in 10% neutral buffered formalin and embedded in paraffin. Thin sections were stained with hematoxylin and eosin. Additional subserial sections from all the paraffin blocks were used for immunohistochemistry. The antibodies used were caspase 3 and caspase 8 (Santa Cruz, Biotecnologies, USA). Immunohistochemistry was performed according to the manufacturer’s instructions. Sections were visualized by treating the slides with diamino-benzidine-tetrahydrochloride.

To determine the levels of apoptotic marker expression, the tumor tissues were assessed in 10 fields by the point-counting technique, using a 100-point grid with a known area (62500 µm^2^ at a 440× magnification) attached to the ocular lens of the microscope [18]. At 400× magnification, the tumor area in each field was calculated according to the number of points hitting the connective tissue as a proportion of the total grid area. Afterward, the number of positive cells within the tumor area was counted [19]. The cell area fraction was determined as the number of positive cells in each field divided by the tumor area. The final results were expressed as percentage mean ± standard deviation (s.d.) of the tumor tissue, with non-coincident microscopic fields. Information about the used immunohistochemical antibodies is in Table S2.

### Optical Microscopy (Hematoxylin/Eosin)

A longitudinal sample of the tumor was selected from each group, processed for paraffin embedding, sectioned (three microns) and stained with hematoxylin-eosin for light microscopy, using an Axiostar plus microscope (Carl Zeiss do Brasil Ltda.). The slides were evaluated by a pathologist with no prior knowledge of the group they belonged to.

### Electron Microscopy

Small fragments of different areas of the tumor from each group were washed and fixed in phosphate-buffered 1% paraformaldehyde and 2% glutaraldehyde (pH 7.3) overnight at 4°C. After fixation, the samples were washed in the same buffer, embedded in molten 2% agar (Merck, Darmstad, Germany) and post-fixed in a mixture of 1% phosphate-buffered osmium tetroxide and 1.5% potassium ferrocyanide for 1 h prior to dehydration in a graded ethanol series and infiltration and embedding in a propylene oxide-Epon sequence (PolyBed 812, Polysciences, Warrington, PA, USA). Thin sections were cut using a diamond knife on an ultramicrotome (Sorvall MT2, Newton, MA, USA) and mounted on uncoated 200-mesh copper grids (Ted Pella, Redding, CA, USA) before staining with uranyl acetate and lead citrate. The samples were viewed using a transmission electron microscope (TEM) (EM 10, Zeiss, Germany) at 60 kV.

### *In situ* Detection of Apoptotic Cells

For the *in situ* detection of apoptosis at the level of a single cell, we used an apoptotic assay of the deoxynucleotidyl transferase (TdT) method of end labeling (TUNEL) (Boehringer Mannheim, Mannheim, Germany) [20], [21]. Thick paraffin sections (4 to 6 µm) were layered on glass slides, deparaffinized with xylene, and rehydrated with graded dilutions of ethanol in water. The slides were washed 4 times with double-distilled water for 2 min and immersed in TdT buffer (Boehringer Mannheim). Then, TdT (0.3 U/µL) and fluorescein-labeled dUTP in TdT buffer were added to cover the section and the samples were incubated in a humid atmosphere at 37°C for 60 min. For negative controls, TdT was eliminated from the reaction mixture. The sections were then incubated with an antibody specific for fluorescein conjugated to peroxidase. The stainings were visualized with a substrate system in which nuclei with DNA fragmentation stained brown. The reaction was terminated by washing the sections twice in PBS. The nuclei without DNA fragmentation stained black as a result of counterstaining with hematoxylin.

### Western Blotting

To detect alterations in protein levels, tumor tissues were treated with or without BNCT. Total cell lysates were obtained with Laemmli Buffer (10% SDS, 0.0625 M Tris-HCl pH 6.8, 10% glycerol, and 5% 2-beta-mercaptoethanol) extraction. Thirty µg of total protein were subjected to electrophoresis in 15% gradient SDS gels under reducing conditions, and subsequently transferred to polyvinylidene diﬂuoride (PVDF) membranes (Hybond-P, Amersham Pharmacia Biotech, Piscataway, NJ, USA). The membranes were incubated with the following antibodies: α-tubulin; Bax; Bcl-2; caspase 3; caspase 7; caspase 8 and caspase 9. Protein bands were detected by enhanced chemiluminescence system ECL (Amersham Pharmacia Biotech). Information about the used western blotting antibodies is in Table S3.

### Statistical Analysis

Values are expressed as the mean ± standard deviation. The data were analyzed using one-way analysis of variance (ANOVA), and significant mean differences were determined using multiple comparisons by the Tukey-Kramer test at p<0.05. Significant differences between the control and treated groups are indicated as *p<0.05, **p<0.01 and ***p<0.001.

For qRT-PCR, statistical tests were evaluated using the JMP Version 5.1 statistical computer program (SAS Institute Inc., Cary, NC). Since assumptions for a parametric test were not valid (Kolmogorov-Smirnov, p<0.05), all data were evaluated by Kruskal-Wallis analysis of variance and the Mann-Whitney U test as a multiple comparison method. Statistical significance was set at probability levels of <0.05.

## Results and Discussion

### BNCT Induces Antiproliferative Effects on Melanoma Cells

The proliferation of B16F10 melanoma cells was evaluated by Ki67 protein expression. Ki67 is used as a marker of cell proliferation in solid tumors [22]. It is a nuclear antigen synthesized throughout the cell cycle, except at the G0 and early G1 phases [23]. Increased proliferative activity of tumor cells is also associated with malignancy and is an important prognostic marker in many human cancers. Ki67 protein is widely used as a marker to evaluate cell proliferation.

**Figure 1:**
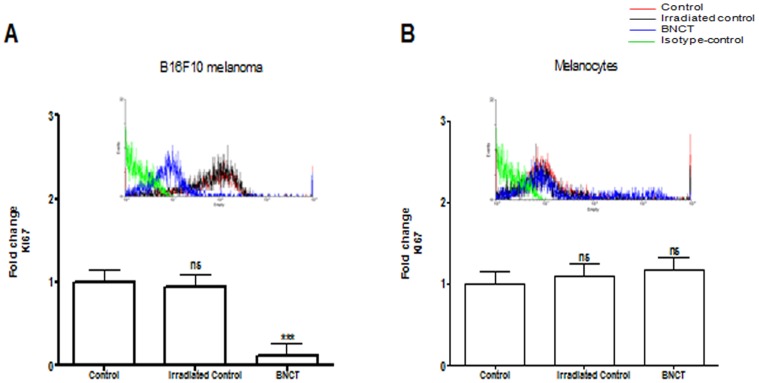
Expression of Ki67 in B16F10 melanoma cells and normal melanocytes (mean ± s.d.) determined by flow cytometry. (A) Ki67 expression in B16F10 melanoma cells after BNCT treatment and neutron irradiation alone (irradiated control) compared to cells without any treatment (control). (B)Ki67 expression in normal melanocytes after BNCT treatment and neutron irradiation alone (irradiated control) compared to cells without any treatment (control). Cells incubated with FITC-conjugated isotype-specific antibodies were used as negative controls. ns: not significant compared to control. *p<0.05; **p<0.01; ***p<0.001 compared to control.

In B16F10 melanoma cells, Ki67 expression was significantly reduced after BNCT treatment, without affecting normal melanocytes. Tumor and normal cells treated only with irradiation did not show significant differences in proliferation to that of control (Figure 1). These findings are in concordance with previous studies demonstrating that a decrease in cyclin D1 induces cell cycle arrest only in tumor cells (murine and human melanoma) after BNCT treatment [24], [10].

### ECM Changes in Melanoma Cells Subjected to BNCT

Interactions between cells and the ECM are crucial for cell behavior, growth and death [25]. The detachment of adherent cells from the ECM can induce apoptosis almost immediately, a process known as anoikis [26].

**Figure 2:**
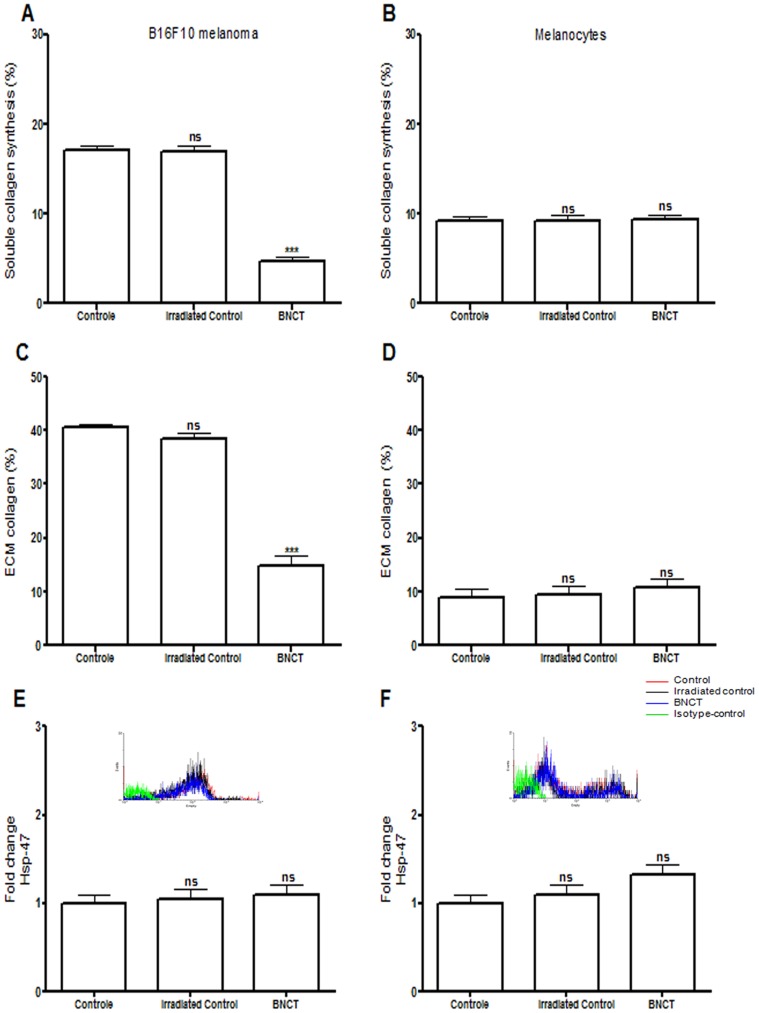
Determination of collagen-related markers in B16F10 melanoma cells and normal melanocytes (mean ± s.d.). (A) Synthesis of soluble collagen in B16F10 melanoma cells after BNCT treatment and neutron irradiation alone (irradiated control) compared to cells without any treatment (control). (B) Synthesis of soluble collagen in normal melanocytes after BNCT treatment and neutron irradiation alone (irradiated control) compared to cells without any treatment (control). (C) Expression of ECM collagen in B16F10 melanoma cells after BNCT treatment and neutron irradiation alone (irradiated control) compared to cells without any treatment (control). (D)Expression of ECM collagen in B16F10 melanoma cells after BNCT treatment and neutron irradiation alone (irradiated control) compared to cells without any treatment (control). (E) Expression of Hsp47 in B16F10 melanoma cells after BNCT treatment and neutron irradiation alone (irradiated control) compared to cells without any treatment (control). (F)Expression of Hsp47in normal melanocytes after BNCT treatment and neutron irradiation alone (irradiated control) compared to cells without any treatment (control). Cells incubated with FITC-conjugated isotype-specific antibodies were used as negative controls. ns: not significant compared to control. *p<0.05; **p<0.01; ***p<0.001 compared to control.

After BNCT, soluble collagen synthesis and ECM collagen were quantified by picrosirius staining. In B16F10 melanoma, control cells showed that approximately 20 µg/10^6^ cells expressed soluble collagen, whereas in BNCT-treated cells, this was seen in less than 5 µg/10^6^ cells (Figure 2A). There were no changes in the secretion of soluble collagen in melanocytes (Figure 2B). The collagen present in the ECM was also significantly reduced after BNCT treatment in melanoma cells (Figure 2C), but did not change in melanocytes (Figure 2D). Soluble collagen and ECM collagen were also reduced in human melanoma, while apoptosis was induced after BNCT treatment [6].

Heat shock proteins (hsp) are ubiquitous and known to be expressed in all organisms [27]. Hsp47 possesses an integral role in procollagen biosynthesis [28]. There was no alteration in Hsp47 expression after BNCT treatment (Figure 2E and F) in B16F10 melanoma cells and melanocytes, indicating that the observed modifications in collagen did not correlate with this marker.

### Apoptosis Induction is Triggered by Intrinsic and Extrinsic Pathways in BNCT-treated Melanoma Cells

**Figure 3:**
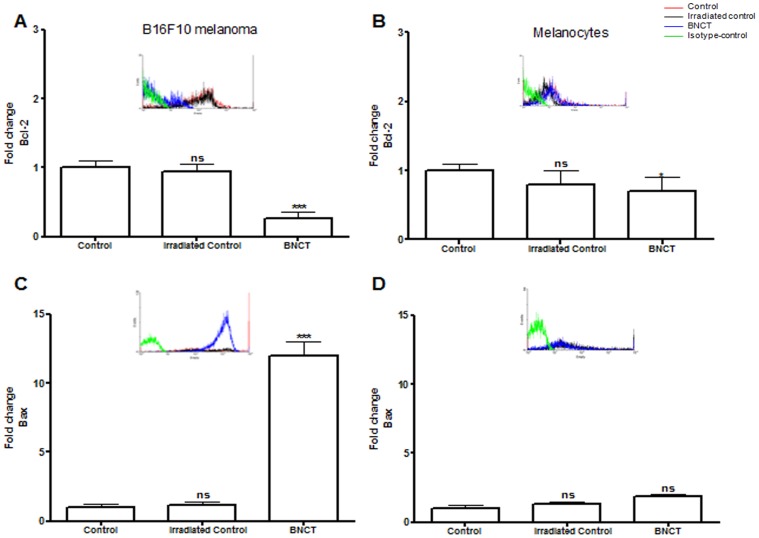
Expression of intrinsic apoptotic markers in B16F10 melanoma cells and normal melanocytes (mean ± s.d.) measured by flow cytometry. (A) Expression of Bcl-2 in B16F10 melanoma cells after BNCT treatment and neutron irradiation alone (irradiated control) compared to cells without any treatment (control). (B)Expression of Bcl-2 in normal melanocytes after BNCT treatment and neutron irradiation alone (irradiated control) compared to cells without any treatment (control). (C) Expression of Bax in B16F10 melanoma cells after BNCT treatment and neutron irradiation alone (irradiated control) compared to cells without any treatment (control). (D)Expression of Bax in normal melanocytes after BNCT treatment and neutron irradiation alone (irradiated control) compared to cells without any treatment (control).Cells incubated with FITC-conjugated isotype-specific antibodies were used as negative controls. ns: not significant compared to control. *p<0.05; **p<0.01; ***p<0.001 compared to control.

B-cell lymphoma-2 (Bcl-2) and Bcl-2 associated X protein (Bax), members of the Bcl-2 family of proteins, are antiapoptotic and proapoptotic factors, respectively [29], [30].Bax can release cytochrome c after the formation of Bax/Bak hetero-oligomer, whereas Bcl-2 can interact with activator proteins or Bax/Bak, thus sequestering these proteins [31]. Our results indicated that BNCT acts by significantly down-regulating Bcl-2 while up-regulating Bax only in melanoma cells. There was a small decrease in the expression of the anti-apoptotic Bcl-2 in normal melanocytes. Comparing these data with those obtained in melanoma cells, these findings were modestly significant, indicating that BNCT can induce damage to normal cells to a lesser degree (Figure 3A–D). BNCT has also been shown to decrease the expression of Bcl-2 and increase the expression of Bax in other tumor cells, such as glioma [32]. However, these markers were not affected in an oral cancer model treated by BNCT, as previously described [33].

**Figure 4:**
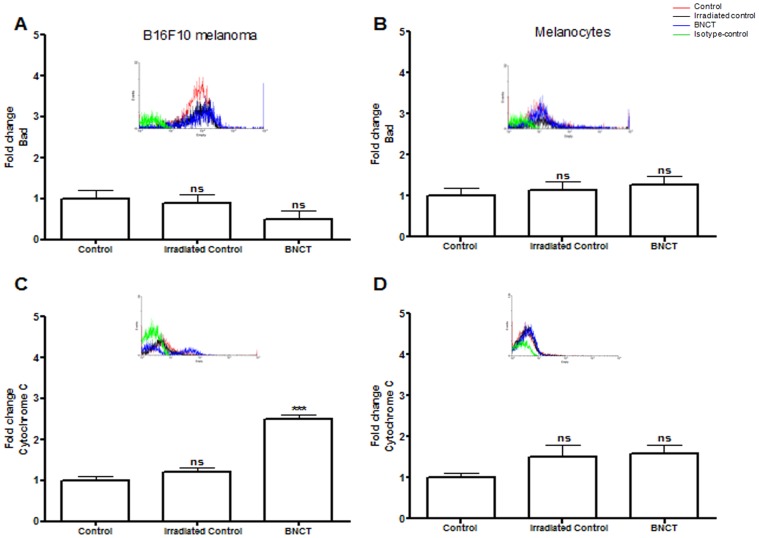
Expression of intrinsic apoptotic markers in B16F10 melanoma cells and normal melanocytes (mean ± s.d.) measured by flow cytometry. (A) Expression of Bad in B16F10 melanoma cells after BNCT treatment and neutron irradiation alone (irradiated control) compared to cells without any treatment (control). (B)Expression of Bad in normal melanocytes after BNCT treatment and neutron irradiation alone (irradiated control) compared to cells without any treatment (control).(C) Cytochrome c expression in B16F10 melanoma cells after BNCT treatment and neutron irradiation alone (irradiated control) compared to cells without any treatment (control). (D)Expression of cytochrome c in normal melanocytes after BNCT treatment and neutron irradiation alone (irradiated control) compared to cells without any treatment (control). Cells incubated with FITC-conjugated isotype-specific antibodies were used as negative controls. ns: not significant compared to control. *p<0.05; **p<0.01; ***p<0.001 compared to control.

Bax and Bcl-2-associated death promoter (Bad) induce cell death [34], which could directly regulate the release of mitochondrial factors involved in apoptosis [35]. BNCT did not alter the expression of Bad in both melanoma cells and melanocytes (Figure 4A and B), suggesting that the induction of apoptosis in the mitochondria is only regulated by Bcl-2/Bax.

Cytochrome c is an electron transport protein found in the mitochondria. It is released into the cytoplasm during the early stages of apoptosis, prior to caspase activation, DNA fragmentation and loss of membrane potential [35]. Cytochrome c release was observed after BNCT treatment in melanoma cells (p<0.001) and at a lower rate in melanocytes (Figure 4C and D) due to a minor boron uptake in normal cells [36]. The findings in melanoma cells subjected to BNCT can be justified by the translocation of cytochrome c from the cytosol to the mitochondria by BH3-domain-containing pro-apoptotic proteins (such as Bax) or by mitochondrial-permeability transition [6], [10].

The cellular response to tumor necrosis factor (TNF-α) is mediated through its interaction with the TNF-R1 and TNF-R2 receptors, which results in the activation of pathways that promote cell survival or apoptosis, depending on the cell type and the biological context. Activation of kinase pathways (including JNK, ERK, p38 MAPK and NF-kB) promotes cell survival, while activation of TNF-α-mediated caspase 8 leads to programmed cell death [37], [38].

**Figure 5:**
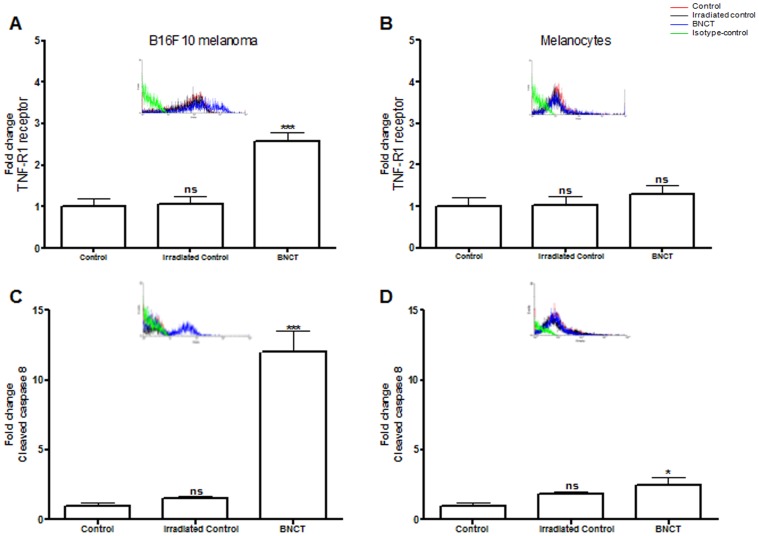
Expression of extrinsic apoptotic markers in B16F10 melanoma cells and normal melanocytes (mean ± s.d.) determined by flow cytometry. (A) Expression of TNF-R1 in B16F10 melanoma cells after BNCT treatment and neutron irradiation alone (irradiated control) compared to cells without any treatment (control). (B)Expression of TNF-R1 in normal melanocytes after BNCT treatment and neutron irradiation alone (irradiated control) compared to cells without any treatment (control). (C) Expression of cleaved caspase 8 in B16F10 melanoma cells after BNCT treatment and neutron irradiation alone (irradiated control) compared to cells without any treatment (control). (D)Expression of cleaved caspase 8 in normal melanocytes after BNCT treatment and neutron irradiation alone (irradiated control) compared to cells without any treatment (control). Cells incubated with FITC-conjugated isotype-specific antibodies were used as negative controls. ns: not significant compared to control. *p<0.05; **p<0.01; ***p<0.001 compared to control.

The extrinsic apoptotic pathway was also activated by BNCT in melanoma cells. TNF receptor expression increased, in addition to caspase 8 cleavage in these tumor cells. None of these cellular markers were affected in melanocytes (Figure 5). These apoptotic markers did change in the cells that were only irradiated (irradiated control) compared to the control.

Cleaved caspase 8 activates molecules as well as downstream caspases such as caspase 3 [39]. The intrinsic and extrinsic pathways are not completely independent. In fact, in some cells, activation of caspase 8 results in the activation of the mitochondrial pathway by Bid cleavage, generating a truncated fragment known as truncated Bid (tBid). tBid can permeabilize the mitochondria, resulting in mitochondrial outer membrane permeabilization [40]. As a final result, caspase 3 cleavage occurs after BNCT treatment in murine and human melanoma cells [24], [6] and in other tumor cells such as undifferentiated thyroid carcinoma [16].

### BNCT Induces *in vivo* Apoptosis in Melanoma

**Figure 6:**
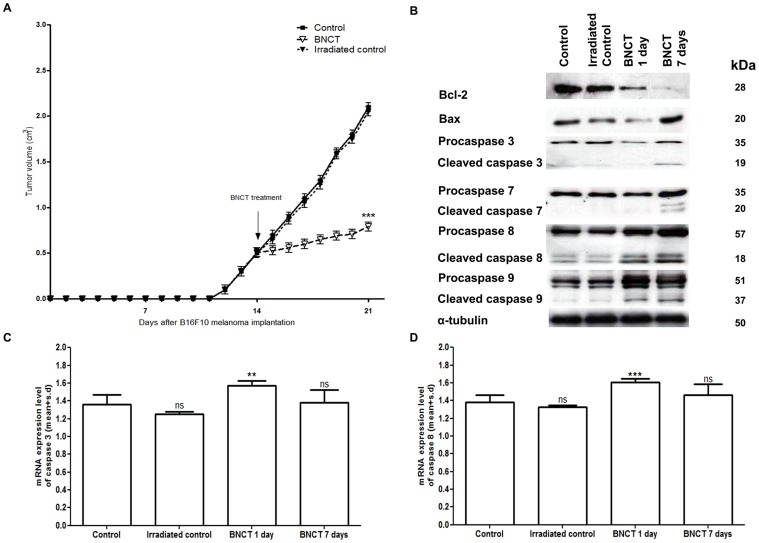
Melanoma cell death after BNCT. (A) Tumor volume of mice bearing B16F10 melanoma during twenty-one days. On the fourteenth day after B16F10 melanoma implantation, the BNCT group received BPA and was irradiated with thermal neutrons. The mice were sacrificed and analyzed 1 or 7 days after BNCT treatment. (B) Western blotting analyses of caspase 3, 7, 8, 9, Bcl-2 and Bax in melanoma tissue. (C, D) mRNA expression levels of caspase 3 and 8, respectively, quantified by RT-PCR.

B16F10 melanoma-bearing mice that had undergone BNCT showed a significantly reduced tumor volume compared to control and the irradiated control groups. on day seven, the mice not treated with BNCT showed about 2.3 cm^3^ of tumor volume, while the BNCT-treated mice exhibited only 0.8 cm^3^ (Figure 6A).

The BNCT-treated mice analyzed after 1 or 7 days of treatment (BNCT 1 day and BNCT 7 day groups) showed a decreased expression of the anti-apoptotic protein Bcl-2 and an increased expression of the pro-apoptotic protein Bax. One day after BNCT, cleaved caspases 8 and 9 were observed. Meanwhile, seven days after BNCT, cleaved caspases 3, 7, 8 and 9 were noted (Figure 6B). This is due to the fact that the first effect of BNCT is necrosis at the site of irradiation (during the first instant), with apoptosis occurring after a certain time (in this case, after 7 days). Gene expression of caspase 3 and 8 was significantly elevated (p<0.01) one day after BNCT (Figure 6C and D). This increase was not found after 7 days of BNCT because there was a substantial presence of these cleaved proteins.

**Figure 7:**
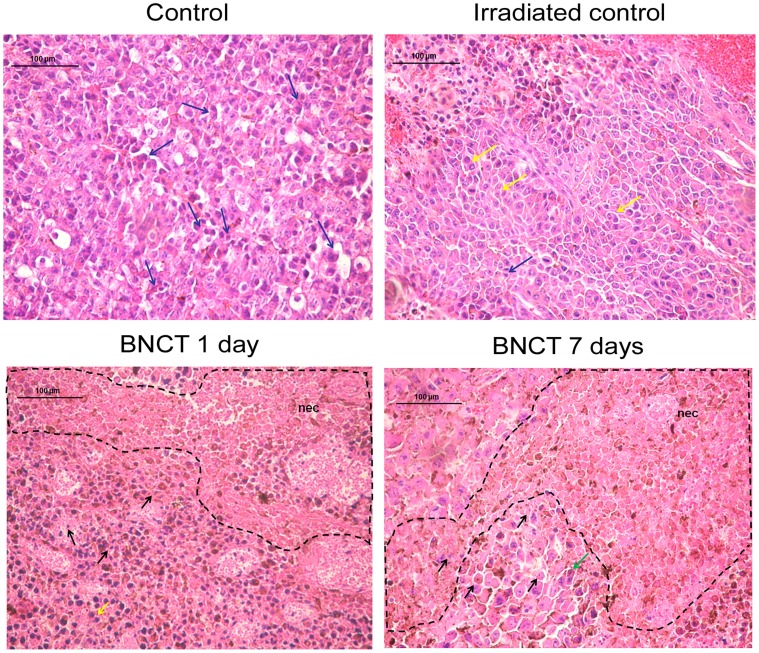
Hematoxylin and eosin-stained sections of malignant melanoma in control, irradiated control, BNCT 1 and BNCT 7 days groups. In the control and irradiated groups, malignant melanoma cells were preserved and composed of large cells with atypical nuclei and abundant cytoplasm. Normal mitosis (blue arrows) and aberrant mitosis (yellow arrows) were both observed. Necrosis was absent in both groups of melanoma. By contrast, extensive necrosis (nec), pycnotic nuclei (black arrows) and acidophilic cytoplasm (green arrows) were present in the malignant melanoma of BNCT 1 and BNCT 7 day groups. Furthermore, the BNCT groups also presented aberrant mitosis.

Hematoxylin and eosin-stained sections revealed malignant melanoma with preserved cells, atypical nuclei and abundant cytoplasm in the control and irradiated control groups. There was the presence of normal and aberrant mitosis. These findings are characteristic hallmarks of proliferative tumor cells [41], [42]. On the other hand, these characteristics were not found in the BNCT groups, which presented necrotic areas, pycnotic nuclei and acidophilic cytoplasm in malignant melanoma after 1 and/or 7 days of BNCT (Figure 7).

**Figure 8:**
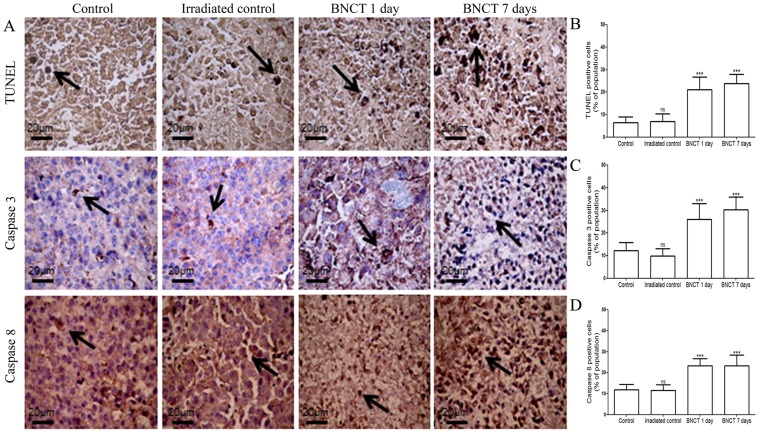
TUNEL, caspase 3- and caspase 8-stained sections and melanoma morphometric analysis of control, irradiated control, BNCT 1 day and 7 days groups. (A) In control and irradiated groups (×400), apoptotic melanoma cells, i.e., those stained with TUNEL, caspase 3 and 8 (arrow), were sparse. In BNCT 1 and 7 day groups (×400), many melanoma cells were in apoptosis. Graphic plots show an increase in apoptotic melanoma cells as determined by (B) TUNEL, (C) caspase 3 and (D) caspase 8 staining in BNCT 1 and 7 day groups. All results are expressed as mean ± s.d. ns: not significant compared to control. *p<0.05; **p<0.01; ***p<0.001 compared to control.

Through quantitative comparison of the apoptotic rates among the four groups of melanoma tissue using TUNEL, we found that the percentage of cells undergoing apoptosis in tissues following 1 and 7 days of BNCT was greater than that of control or irradiated control (Figure 8A and B). Furthermore, we performed immunostaining associated with apoptosis, using anti-caspase 3 and anti-caspase 8 antibodies. Analysis of the numbers of caspase 3- and 8-positive cells (brown) showed that BNCT after 1 and/or 7 days was clearly more potent in eliciting tumor cell apoptosis than control or irradiated control (Figure 8C and D).

**Figure 9:**
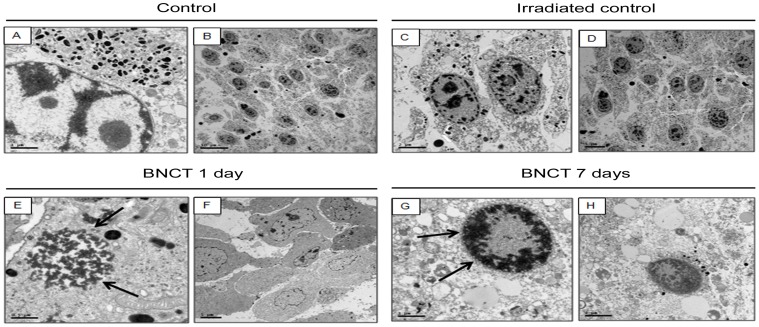
Electron microscopy of melanoma cells from control, irradiated control, BNCT 1 and 7 days groups. (A,C) Detail of the preserved chromatin in the nuclei of melanoma cells from control and irradiated control groups. (B, D) Illustration of the high density population. By contrast, in panels (E, G, H), melanoma cells show a markedly condensed chromatin close to the nuclear membrane (arrows) 1 and 7 days after BNCT. (F) Decrease in cell density after BNCT. The organelles inside the melanoma cells appeared to be degenerated in both BNCT groups.

Electronic microscopy of control and irradiated control showed preserved chromatin in the nuclei of melanoma cells, high cell population densities and exacerbated amount of melanosomes, whereas BNCT-treated samples displayed condensed chromatin close to the nuclear membrane, cell density decreases and degenerated organelles (Figure 9). These results, together with the biochemical features [41], [42] demonstrated above, confirmed that BNCT-treated cells and tumor tissues underwent apoptosis.

These data confirm apoptosis after BNCT, as also noted by Masunaga [20], [21], Wang [32] and Fujita [43], who observed apoptosis *in vitro* and *in vivo* in mouse lymphoma, glioma and oral squamous cell carcinoma, respectively. Some authors report that BNCT apoptosis may be specific to some tumor types, for example, Aromando [33] and Kamida [44], who studied hamster cheek pouch tumor and human oral squamous cell carcinoma xenografts, respectively.

BNCT *in vivo* melanoma treatment show many positive characteristics, as well as presenting few alterations in normal tissues [6]. Thus, this therapy could be an attractive tool for treating this neoplasia. The mode of action of BNCT in melanoma cells could involve Bcl-2 down-regulation, Bax up-regulation, caspase 9 cleavage and cytochrome c release, inducing apoptosis through the mitochondrial pathway. In addition, there were increases in TNF-R1 expression and caspase 8 cleavage after BNCT, showing that apoptosis induced by BNCT can also be mediated through extrinsic pathways. In this way, these findings confirm apoptosis by both pathways in BNCT-treated melanoma (*in vitro* and *in vivo*).

### Conclusions

BNCT inhibited melanoma proliferation, altered ECM collagen synthesis and induced apoptosis by regulating Bcl-2/Bax expression, as well as increasing the levels of TNF receptor and cleaved caspases 3, 7, 8 and 9 in melanoma cells. These results suggest that multiple pathways related to cell death and cell cycle arrest are involved in the treatment of melanoma by BNCT.

## Supporting Information

Table S1 Antibodies used in flow cytometry experiments.
(DOC)

Table S2 Antibodies used in immunohistochemistry experiments.
(DOC)

Table S3 Antibodies used in western blots experiments.
(DOC)
