# Supplementary material for: Apoptosis through Bcl-2/Bax and Cleaved Caspase Up-Regulation in Melanoma Treated by Boron Neutron Capture Therapy
Source: PLoS One. 2013 Mar 20;8(3):e59639. doi: 10.1371/journal.pone.0059639 (PMC3603877; doi:10.1371/journal.pone.0059639)
Supplement: Table S1 — Antibodies used in flow cytometry experiments. (DOC) [file pone.0059639.s001.doc]

**Supporting Information**

**Apoptosis through Bcl-2/Bax and cleaved caspase up-regulation in melanoma treated by Boron Neutron Capture Therapy**

Fernanda Faião-Flores, Paulo Rogério Pinto Coelho, João Dias Toledo Arruda-Neto, Silvya Stuchi Maria-Engler, Manoela Tiago, Vera Luiza Capelozzi, Ricardo Giorgi, and Durvanei Augusto Maria

TABLE S1
